# pH-Responsive fluorescent supramolecular nanoparticles based on tetraphenylethylene-labelled chitosan and a six-fold carboxylated tribenzotriquinacene

**DOI:** 10.3762/bjoc.19.45

**Published:** 2023-05-08

**Authors:** Nan Yang, Yi-Yan Zhu, Wei-Xiu Lin, Yi-Long Lu, Wen-Rong Xu

**Affiliations:** 1 Key Laboratory of Advanced Materials of Tropical Island Resources of Ministry of Education, Hainan Provincial Key Laboratory of Fine Chemistry, School of Chemical Engineering and Technology, School of Science, Hainan University, Haikou 570228, PR China

**Keywords:** chitosan, nanoparticle, tetraphenylethylene, tribenzotriquinacene

## Abstract

We synthesized a new tetraphenylethylene-modified chitosan bioconjugate, **CS-TPE**, that shows the aggregation-induced emission effect. It can self-assemble into fluorescent polymeric nanoparticles in an aqueous solution at pH 5.3 either alone or with the water-soluble bowl-shaped six-fold carboxylated tribenzotriquinacene derivative **TBTQ-C_6_** via host–guest binding. The spherical nanoparticles formed by **CS-TPE** amphiphiles or **TBTQ-C_6_/CS-TPE** supra-amphiphiles disintegrated under alkaline stimulation at pH 10.4 and the dispersion of the aggregates after the collapse in the presence of **TBTQ-C_6_** was greatly improved. In addition, the fluorescence of **CS-TPE** was significantly enhanced by introducing **TBTQ-C_6_**, and remained relatively stable with variations in pH for both **CS-TPE** and **TBTQ-C_6_**/**CS-TPE**. Such pH-responsive supramolecular spherical nanoparticles with stable fluorescence emission based on **CS-TPE** or **TBTQ-C_6_/CS-TPE** may find applications in various fields, including the development of visual oral drug delivery systems.

## Introduction

Stimuli-responsive assemblies in aqueous media have drawn extensive attention owing to their potential applications in biomedicine, chemical sensing and as catalysts [1–2]. It is possible to regulate the formation and breakdown of supramolecular structures using external stimuli, such as pH, light, temperature, enzymes, and competing reagents [3–4]. Among these stimuli, pH response is of particular interest in the construction of supramolecular systems for intelligent drug delivery because of the presence of pH gradients in the microenvironment of organs, tissues, and cell organelles [5]. In the past years, pH-responsive and other stimuli-responsive supramolecular systems derived from water-soluble macrocycles, including cyclodextrins (CDs), calix[*n*]arenes (CXs), cucurbiturils (CBs), and pillararenes have been widely studied [2,6–8]. Similar to those macrocyclic acceptors, tribenzotriquinacene (TBTQ) derivatives, a class of versatile host molecules with a bowl-shaped *C*_3v_-symmetric skeleton, are capable of effectively encapsulating guest molecules [9–15]. In recently published studies [16–17], we have successfully synthesized two water-soluble hexacarboxylated tribenzotriquinacene derivatives, 2,2',2'',2''',2'''',2'''''-((12d-methyltribenzotriquinacene-2,3,6,7,10,11-hexayl)hexakis(oxy))hexaacetate (**TBTQ-C_6_**) and 2,2',2'',2''',2'''',2'''''-((((12d-methyltribenzotriquinacene-2,3,6,7,10,11-hexayl)hexakis(oxy))hexakis(methylene))hexakis(1*H*-1,2,3-triazole-4,1-diyl))hexaacetate (**TBTQ-CB6**), which act as host molecules to bind to guest molecules to construct pH-responsive supramolecular vesicles and molecule-scale drug carriers, respectively. Both of them exhibited pH-responsive properties, with the host–guest binding disintegrating when the pH of the solution is adjusted to 5.5 and the binding regenerated when the pH is restored to 7.4. These stimulus-responsive supramolecular vesicles and molecule-scale drug carriers are considered to have potential for cancer drug delivery. However, such supramolecular systems are not suitable for oral administration, which is a convenient and patient-preferred method of drug delivery, especially for patients with chronic conditions requiring frequent doses of medication [18]. The aggregates may have collapsed in an acidic environment in the stomach (pH 1.5–3.5) before reaching the intestines (pH 7.0–8.5) and releasing their contents [19]. Therefore, it is of great interest to develop self-assembled systems based on TBTQ with fluorescent imaging modules that are stable in acidic environments and collapse/swell in alkaline environments.

**Scheme 1:**
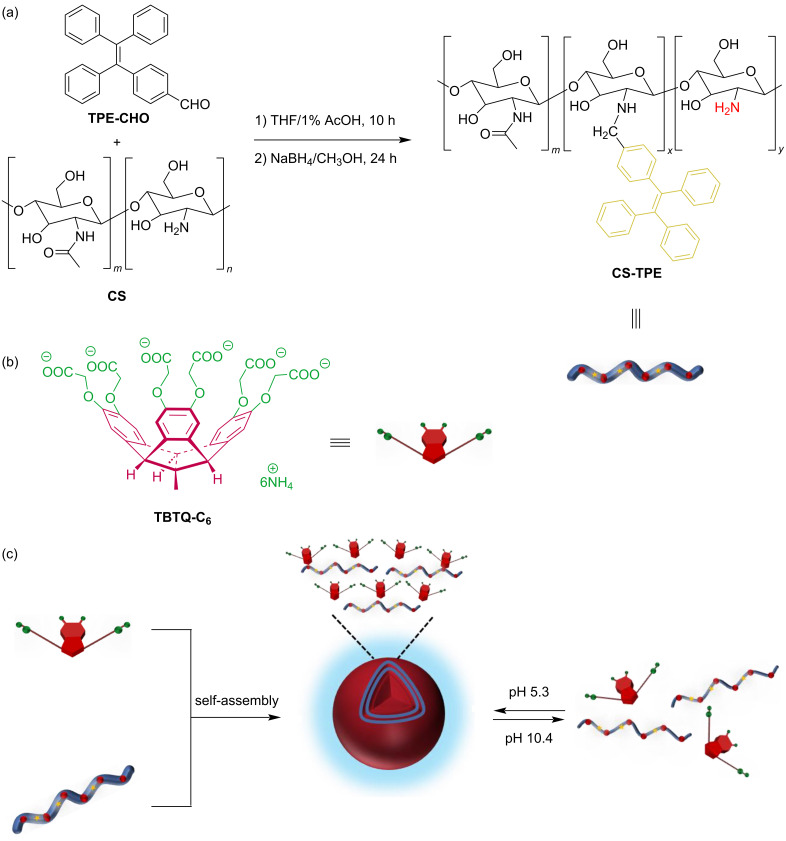
(a) Synthesis route to **CS-TPE**. (b) Structure of **TBTQ-C_6_**. (c) Construction of **TBTQ-C_6_**/**CS-TPE** supramolecular nanoparticles and their pH-responsive behaviors.

The development of nanoscale drug delivery systems equipped with synchronized fluorescent imaging modules has attracted significant interest in recent years. However, conventional fluorescent dyes exhibit aggregation-caused quenching (ACQ) when introduced into nanoparticles at high loads, resulting in poor fluorescence imaging quality. In recent years, tetraphenylethylene (TPE)-based dyes have been frequently used to overcome the ACQ problem due to their unique aggregation-induced emission (AIE) character [20–21]. Chitosan (CS) is a natural cationic polysaccharide, which is considered an ideal material for the construction of drug delivery systems due to its nontoxic, biocompatible, biodegradable, and easily functionalized properties [22]. Herein, we designed and synthesized a new chitosan bioconjugate **CS-TPE** with varying amounts of TPE labelling. It can self-assemble into fluorescent polymeric nanoparticles in an aqueous solution at pH 5.3, either alone or as a guest by electrostatic interaction with the **TBTQ-C_6_** host. These nanoparticles were disassembled upon exposure to alkaline environmental stimuli at pH 10.4, and the assembly and disassembly processes were reversible (Scheme 1). The introduction of the **TBTQ-C_6_** host significantly improved the dispersion of the aggregates formed after the collapse of the spherical nanoparticles under alkaline conditions. In addition, the presence of **TBTQ-C_6_** greatly enhanced the fluorescence emission of the nanoparticles and was able to maintain the fluorescence relatively stable at different pH values. Essentially, this work demonstrates the first study involving stimuli-responsive supramolecular polymeric nanoparticles based on **TBTQ-C_6_/CS-TPE** supra-amphiphile, which may have potential applications in oral drug delivery materials and biomarkers.

## Results and Discussion

**Figure 1:**
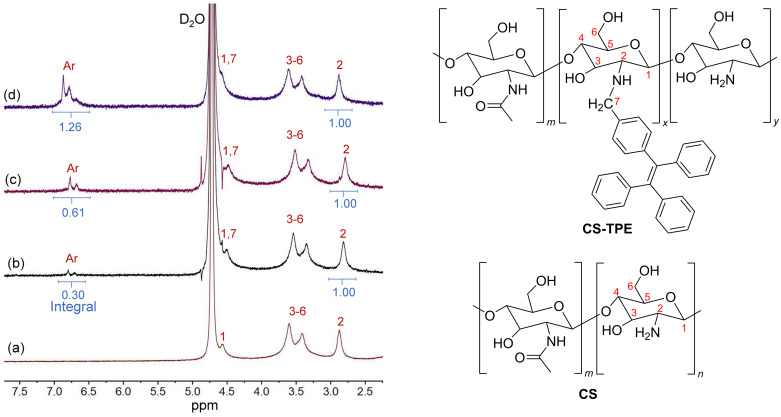
Partial ^1^H NMR spectra (400 MHz, CD_3_COOD/D_2_O, 25 °C) of (a) CS, (b) **CS-TPE-2%**, (c) **CS-TPE-10%**, and (d) **CS-TPE-20%**.

**Synthesis and characterization of CS-TPE.** The host molecule **TBTQ-C_6_** was synthesized as reported in our previously reported method [16]. Three **CS-TPE** bioconjugates with different degrees of fluorescence labelling were synthesized as guests by controlling the feed ratios (*R*_f_) of 4-(1,2,2-triphenylvinyl)benzaldehyde (**TPE-CHO**) [23] to 2 mol %, 10 mol %, and 20 mol %, respectively. They were obtained with chitosan and **TPE-CHO** through an aldimine condensation reaction to generate the corresponding multiple Schiff bases and then reduced with sodium borohydride (Scheme 1a). The structure of **CS-TPE** was characterized by ^1^H NMR spectra and solid-state CP/MAS ^13^C NMR spectra, and the results were found to agree with the proposed structure. For instance, the products display distinct peaks at δ 7.50–6.70 in their ^1^H NMR spectra (Figure 1) and at δ 145–120 ppm in their ^13^C NMR spectra (Figure S1 in Supporting Information File 1), corresponding to the proton resonances of the benzene rings and the carbon resonances of the whole fluorophore, respectively. The appearance of these peaks confirmed that the TPE units were successfully labelled in the CS chain. The degree of fluorescence labelling (DL) of TPE attached to the CS chain was estimated by comparing the integrated areas of H^ar^ and H^2^ in the ^1^H NMR spectra (Figure 1) [24]. Accordingly, the DL at *R*_f_ = 2, 10 and 20 mol % was calculated to be 1.6, 3.2 and 6.6 mol %, respectively, increasing as *R*_f_ increased.

**Figure 2:**
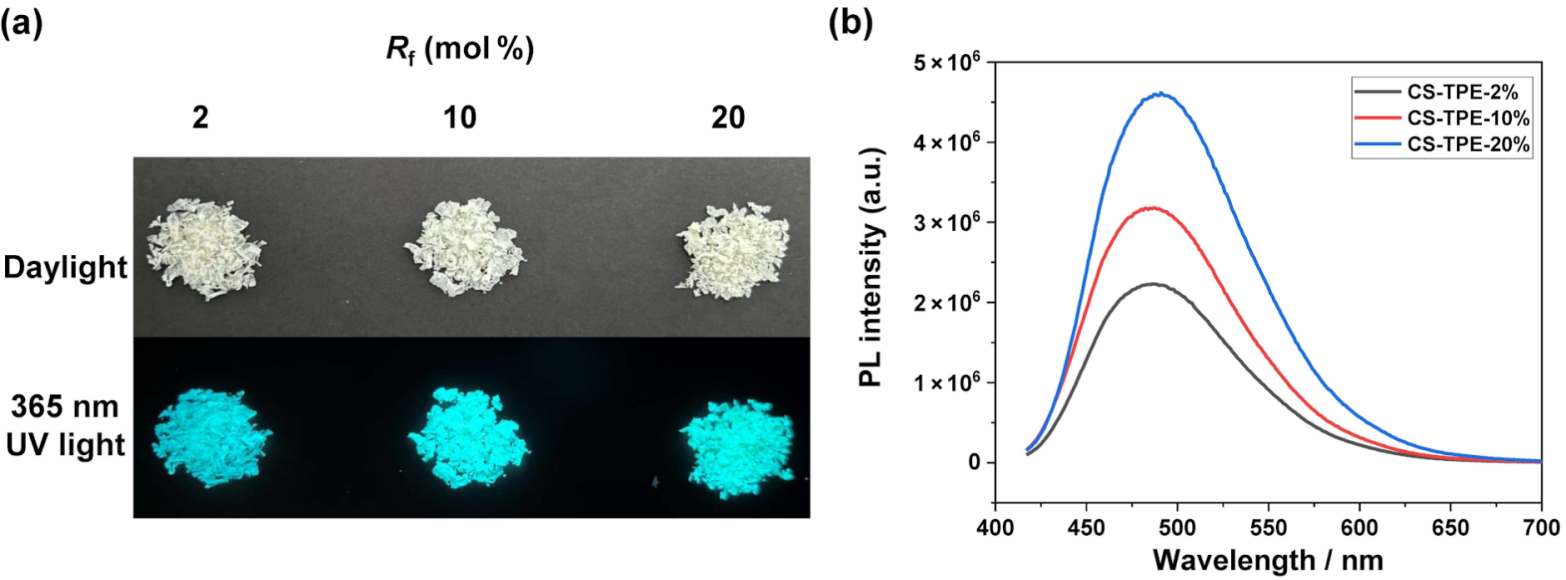
(a) Optical images of **CS-TPE** under daylight (top) and 365 nm UV light (bottom) in the solid state; (b) fluorescence spectra of **CS-TPE** in the solid state (λ_ex_ = 397 nm and λ_em_ = 485 nm).

**Fluorescent properties of CS-TPE in the solid state. CS-TPE** bioconjugates were found to be AIE active, like other TPE-modified derivatives in previous works [25–26]. As shown in Figure 2a, the solid powders of **CS-TPE** exhibit a pale-yellow appearance under normal daylight conditions and emit intense green-blue light when exposed to UV irradiation (365 nm). This phenomenon differs greatly from the fluorescence properties of conventional ACQ fluorophores, which are typically nonfluorescent under UV illumination in the solid state due to powerful self-quenching effects in the aggregates [25]. Strong emission is observed at approximately 485 nm for all **CS-TPE** bioconjugates in the solid state (Figure 2b). A major reason for the emission behavior is the restriction in intramolecular rotation (RIR) of aromatic rotors of the TPE molecules in aggregates, which blocks nonradiative decay channels and enables the formation of radiative transitions, resulting in high fluorescent properties of the aggregates [27].

Additionally, the photoluminescence (PL) intensity is significantly increased with an increased TPE labelling degree.

**Self-assembly behavior of CS-TPE in water.** To investigate the self-assembly behavior of **CS-TPE** in water, the critical aggregation concentrations (CACs) of **CS-TPE** were determined by measuring their optical transmittance at different concentrations (Figure S2, Supporting Information File 1). When the concentration of **CS-TPE** in aqueous solutions at pH 5.3 was increased from 0 to 80 µg/mL, the optical transmission at 293 nm gradually decreased, suggesting the formation of larger-size aggregates in the solution. As indicated by the inflection points in the curve of optical transmittance at 293 nm versus the concentration of **CS-TPE**, the CACs of **CS-TPE** with *R*_f_ = 2, 10 and 20 mol % were determined to be 32 µg/mL, 34 µg/mL and 35 µg/mL, respectively. Furthermore, **CS-TPE** solutions with concentrations above the CAC values exhibited a significant Tyndall effect, suggesting the existence of a large number of nanoparticles. Conversely, solutions with concentrations below the CAC values did not show an apparent Tyndall effect (Figure S3 in Supporting Information File 1), which provides further support for the accuracy of the CAC values.

**Figure 3:**
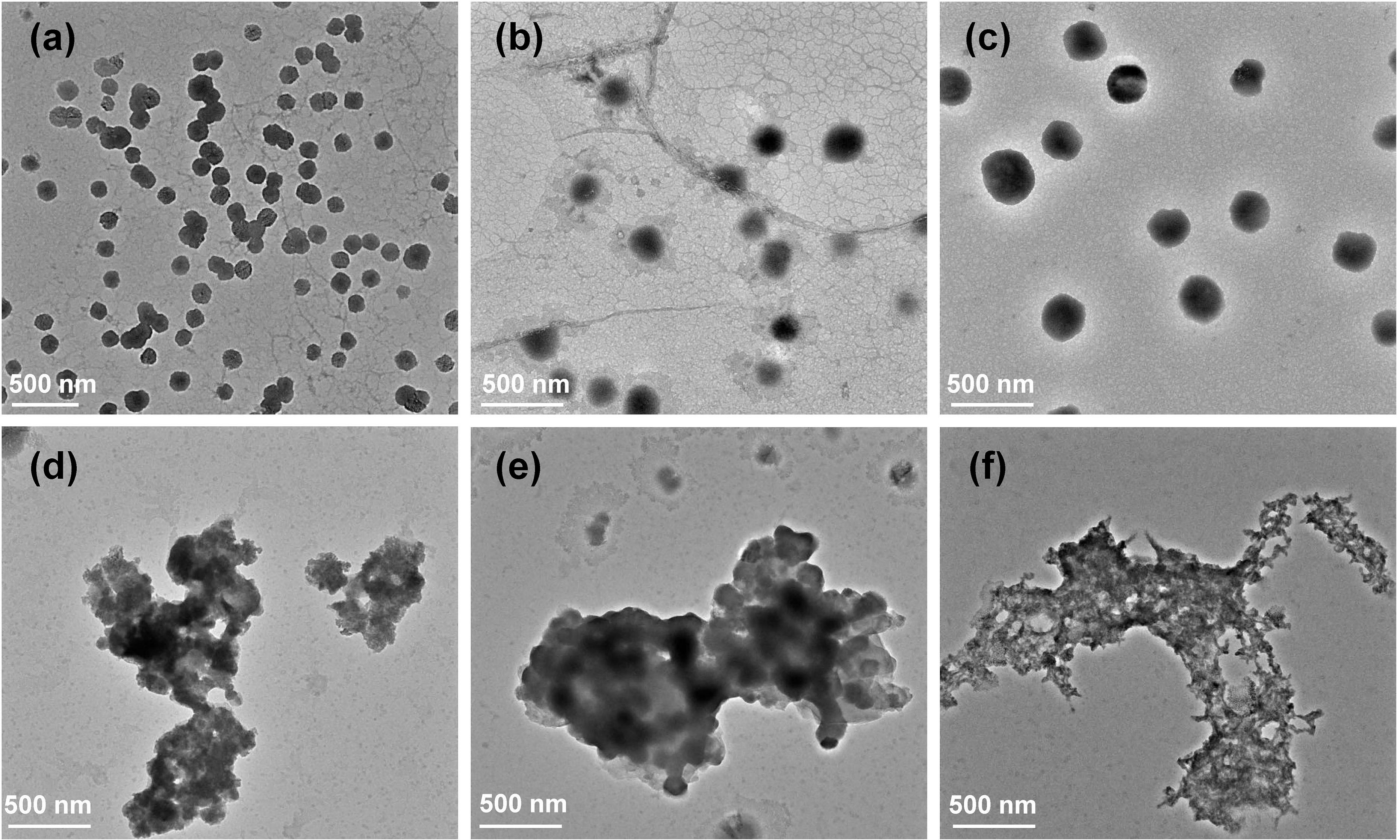
TEM images of (a) **CS-TPE-2%**, (b) **CS-TPE-10%**, and (c) **CS-TPE-20%** assemblies at pH 5.3; TEM images of (d) **CS-TPE-2%**, (e) **CS-TPE-10%**, and (f) **CS-TPE-20%** at pH 10.4 ([**CS-TPE-2%**] = 32 μg/mL), [**CS-TPE-10%**] = 34 μg/mL, [**CS-TPE-20%**] = 35 μg/mL).

Transmission electron microscopy (TEM) was used to observe the morphology of **CS-TPE** aggregates. At pH 5.3, **CS-TPE** with *R*_f_ = 2, 10 and 20 mol % assembled into homogeneous spherical nanoparticles with average diameters of 131 nm, 166 nm and 236 nm, respectively (Figure 3a–c). This assembly behavior is supposed to be caused by the amphiphilic nature of **CS-TPE**, i.e., the hydrophilicity of the protonated amino groups in an acidic environment combined with the hydrophobicity of the TPE units. Their morphologies under alkaline conditions were studied to investigate the effect of pH on the stability of the **CS-TPE** nanoparticles. When the pH of the solution was increased to 10.4, the nanoparticles collapsed and aggregated (Figure 3d–f), which could be attributed to the poor solubility of the samples bearing neutral amino groups of **CS-TPE** under alkaline conditions, leading to the accumulation of molecular chains. The pH-responsive behavior of **CS-TPE** makes it suitable for oral drug delivery. However, the aggregation behavior of **CS-TPE** under alkaline conditions may influence its drug release efficiency. Thus, the development of modified systems with reduced propensity to accumulate should be of interest.

**Figure 4:**
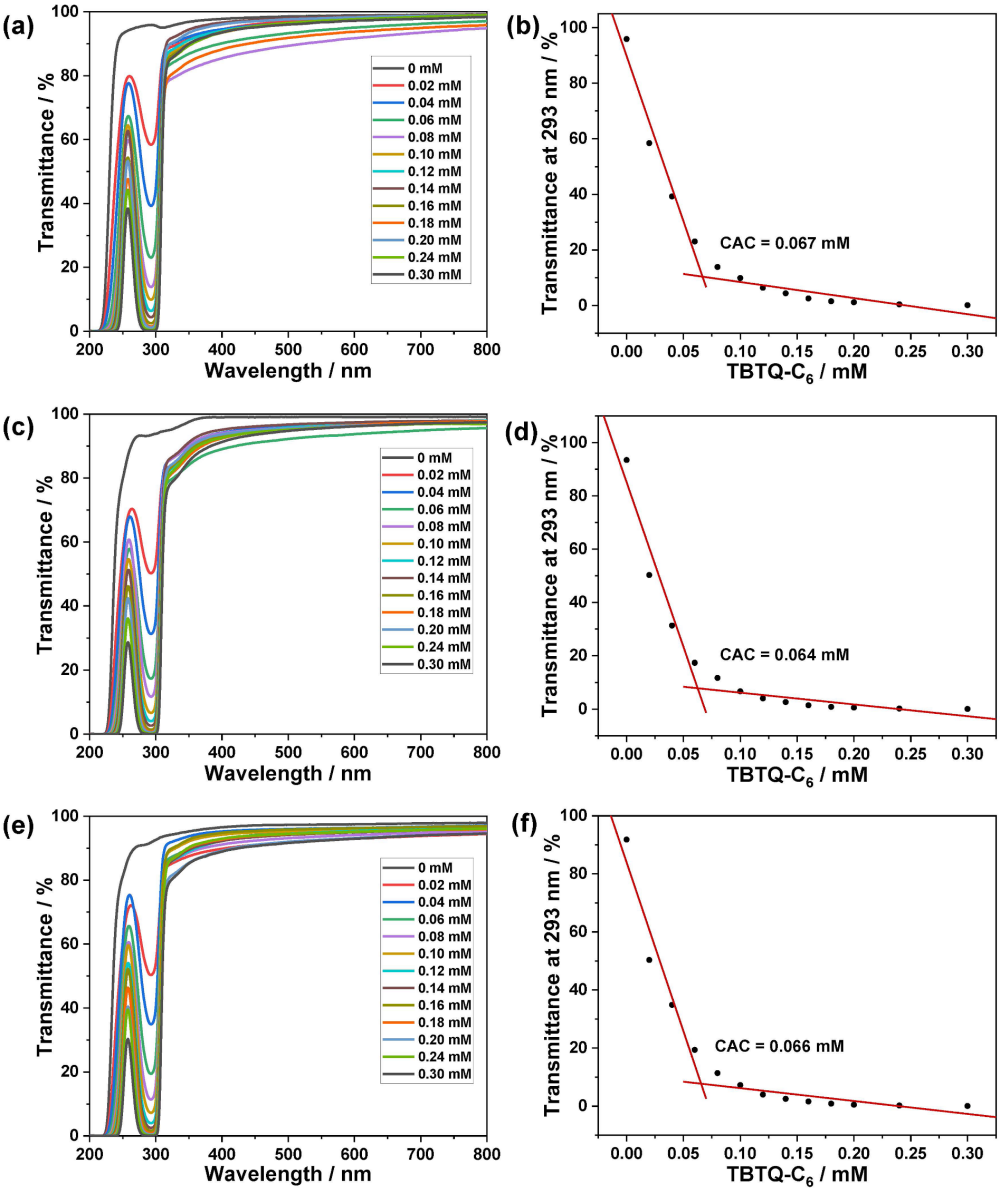
(a, c, e) Optical transmittance and (b, d, f) transmittance as a function of [**TBTQ-C_6_**] at 293 nm of (a, b) **CS-TPE-2%** (10 μg/mL), (c, d) **CS-TPE-10%** (10 μg/mL), and (e, f) **CS-TPE-20%** (10 μg/mL) with varying concentrations of **TBTQ-C_6_** **(**0–0.30 mM) and in aqueous solution at pH 5.3.

**Self-assembly behavior of TBTQ-C_6_/CS-TPE in water.** The anionic bowl-shaped tribenzotriquinacene derivative **TBTQ-C_6_** was incorporated into the polycationic **CS-TPE** to form electrostatically induced host–guest supra-amphiphiles, which further self-assembled into supramolecular nanoparticles in aqueous medium driven by the hydrophobic effect. As displayed in Figure S4 (Supporting Information File 1), free **TBTQ-C_6_** (0.10 mM) showed no Tyndall effect in an aqueous solution. However, when mixed with **CS-TPE** at a concentration of 10 μg/mL, well below their CAC values, clear Tyndall effects were observed, indicating the formation of a large number of nanoparticles in the solution. The CACs of **TBTQ-C_6_** in host–guest complexations were determined by measuring their optical transmittance at a fixed concentration of **CS-TPE** (10 μg/mL) and varying concentrations of **TBTQ-C_6_** **(**0–0.30 mM) [28]. As shown in Figure 4a and 4b, the transmittance of the **TBTQ-C_6_/CS-TPE-2%** complex at 293 nm gradually decreased as the concentration of **TBTQ-C_6_** increased, indicating the formation of larger aggregates. Moreover, the transmittance of the complex at 293 nm displayed a different linear relationship with **TBTQ-C_6_** concentration, with an inflection point at 0.067 mM. Thus, the CAC value of **TBTQ-C_6_** in the presence of **CS-TPE-2%** (10 μg/mL) was determined to be 0.067 mM. Similarly, the CACs of **TBTQ-C_6_** containing **CS-TPE-10%** (10 μg/mL) and **CS-TPE-20%** (10 μg/mL) were measured as 0.064 mM (Figure 4c and 4d) and 0.066 mM (Figure 4e and 4f), respectively.

**Figure 5:**
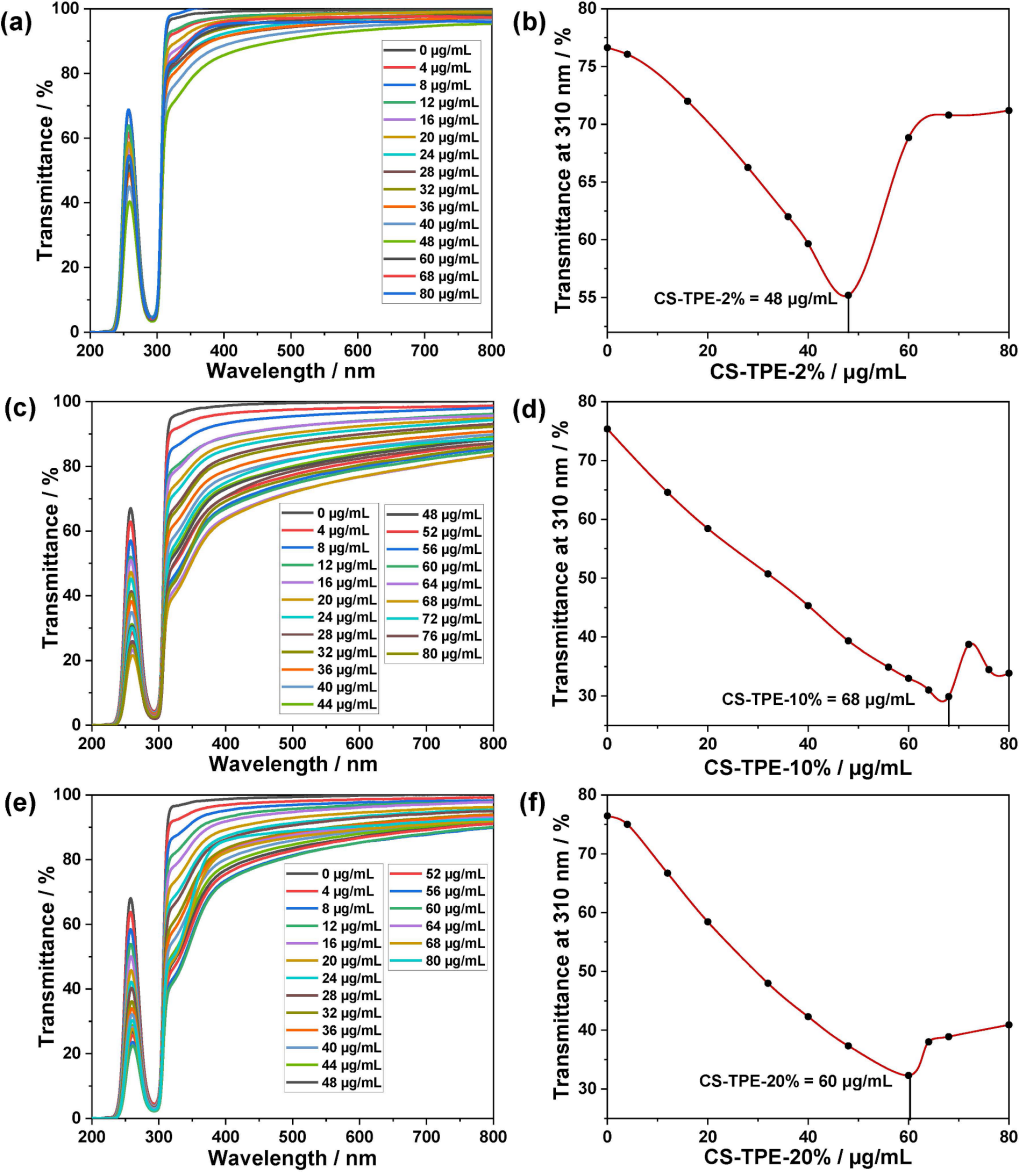
(a, c, e) Optical transmittance and (b, d, f) **CS-TPE** concentration-dependent optical transmittance at 310 nm of **TBTQ-C_6_** (0.10 mM) and varying concentrations (0–80 µg/mL) of (a, b) **CS-TPE-2%**, (c, d) **CS-TPE-10%**, and (e, f) **CS-TPE-20%** in aqueous solution at pH 5.3.

The optimal mixing ratios of **TBTQ-C_6_** and **CS-TPE** were further studied. Under a fixed concentration of **TBTQ-C_6_** (0.1 mM), the transmission at 310 nm initially decreased rapidly and then increased with an increase in **CS-TPE-2%** concentration (Figure 5a and 5b). The rapid decline in transmittance may be attributed to the formation of higher-order complexes between **TBTQ-C_6_** and **CS-TPE-2%** with a tendency toward polymeric supra-amphiphilic assembly. The subsequent increase in transmittance upon addition of an excess of **CS-TPE-2%** was probably caused by the breakdown of the supra-amphiphilic assembly. The transmittance reached its lowest value when the concentration of **CS-TPE-2%** was 48 µg/mL, where a simple inclusion complex formed between **TBTQ-C_6_** and **CS-TPE-2%**. As a result, the optimal mixing ratio for the formation of **TBTQ-C_6_**/**CS-TPE-2%** supra-amphiphilic assembly is 0.10 mM **TBTQ-C_6_** + 48 µg/mL **CS-TPE-2%**. Analogously, the optimal mixing ratios for the formation of **TBTQ-C_6_**/**CS-TPE-10%** and **TBTQ-C_6_**/**CS-TPE-20%** supra-amphiphilic assemblies are 0.10 mM **TBTQ-C_6_** + 68 µg/mL **CS-TPE-10%** (Figure 5c and 5d) and 0.10 mM **TBTQ-C_6_** + 60 μg/mL **CS-TPE-20%** (Figure 5e and 5f), respectively.

**Figure 6:**
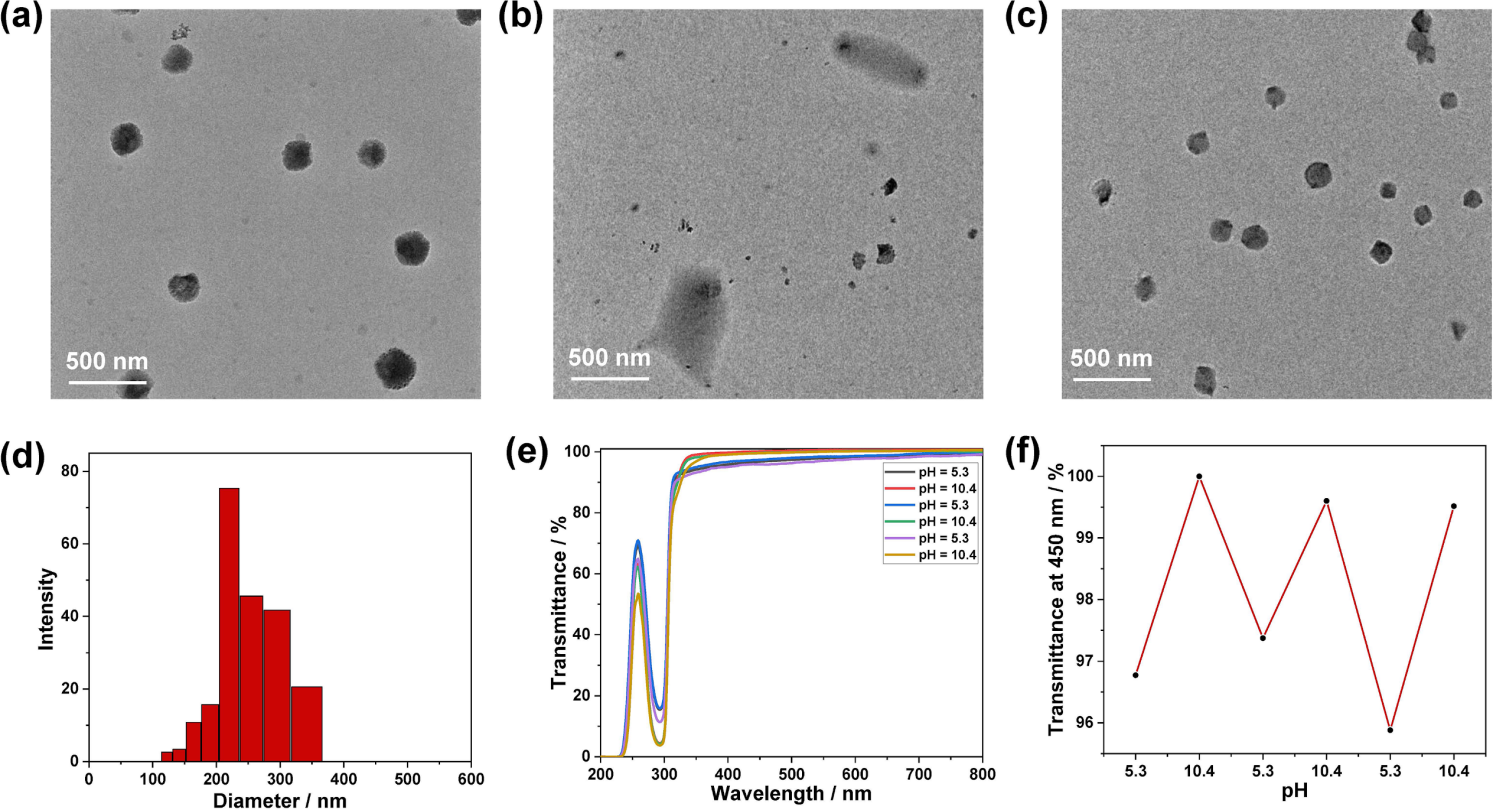
TEM images of (a) **TBTQ-C_6_**/**CS-TPE-10%** in aqueous solution at pH 5.3, (b) after adjustment of the solution (a) to pH 10.4, (c) after readjustment of solution (b) to pH 5.3, (d) DLS data of **TBTQ-C_6_**/**CS-TPE-10%** assembly, (e) optical transmittance and (f) dependence of the optical transmittance at 450 nm of the **TBTQ-C_6_**/**CS-TPE-10%** assembly under acidic and alkaline conditions over several cycles ([**TBTQ-C_6_**] = 0.10 mM, [**CS-TPE-10%**] = 68 μg/mL).

The morphology and size distribution of **TBTQ-C_6_**/**CS-TPE** aggregates were characterized by TEM and dynamic laser scattering (DLS). **TBTQ-C_6_**/**CS-TPE** containing either of the different TPE-loadings all formed uniform spherical nanoparticles with average diameters of 152 nm, 158 nm and 199 nm, respectively, as determined by TEM measurements (Figure 6a and Figures S5a and S6a in Supporting Information File 1). The size distributions of the aggregates measured by DLS ranged from 190 to 396 nm, 122 to 342 nm and 164 to 342 nm, respectively (Figure 6d and Figures S5d and S6d in Supporting Information File 1). As expected, the aggregate sizes measured by DLS were slightly larger than those measured by TEM. This is because TEM probes samples in the dry state, while DLS examines samples in the solvated state, where the solvent molecules are associated with the nanoparticles. The zeta potentials of these three **TBTQ-C_6_**/**CS-TPE** nanoparticles were further measured to determine their surface charge distribution. The measurements revealed that they all had negatively charged surfaces with average zeta potentials of −1.5 mV, −10.9 mV, and −12.2 mV, respectively. These results suggested that **CS-TPE** obtained from higher *R*_f_ could form more stable polymeric supra-amphiphiles with **TBTQ-C_6_** in water at pH 5.3 due to the more pronounced steric hindrance and the stronger charge repulsion on their complex surface [29–30]. Moreover, we propose that **TBTQ-C_6_**/**CS-TPE** supra-amphiphiles connected by electrostatic interactions self-assemble in water layer-by-layer with the outer surface composed of **TBTQ-C_6_**, as illustrated in Scheme 1.

Due to the electrostatic interaction between the host and guest molecules, pH is considered an influential control factor in regulating the assembly and disassembly of supramolecular nanoparticles, as changes in pH affect the protonation degree of the carboxylate functionalities of **TBTQ-C_6_** and the amino groups of **CS-TPE**. Therefore, we investigated the pH-responsive behavior of the **TBTQ-C_6_/CS-TPE** complexes by transmittance and TEM measurements. As displayed in Figure 6e and 6f, the optical transmittance increased when the pH of the solution of **TBTQ-C_6_/CS-TPE-10%** complex increased from 5.3 to 10.4, which indicates that the nanoparticles decomposed at a higher pH value. In turn, the transmittance decreased to the original level when the pH of the solution was readjusted to 5.3. As a result, nanoparticles appeared to regenerate themselves, and this cycle could be repeated several times. The TEM results showed that the spherical nanoparticles were disrupted when the pH increased from 5.3 to 10.4 without forming significant aggregations (Figure 6b) as in the case of **CS-TPS-10%** alone (Figure 3e), and this difference may be attributed to the enhanced water solubility of **CS-TPS-10%** after combining with **TBTQ-C_6_**. Furthermore, the complex reassembled into spherical nanoparticles when the pH of the solution was restored to 5.3 (Figure 6c). These results are consistent with the transmittance measurements, and similar phenomena were observed for the **TBTQ-C_6_/CS-TPE-2%** (Figure S5, Supporting Information File 1) and **TBTQ-C_6_/CS-TPE-20%** (Figure S6, Supporting Information File 1) complexes. Thus, **TBTQ-C_6_/CS-TPE** nanoparticles exhibited a high pH response as well as reversible assembly and disassembly capabilities.

**Figure 7:**
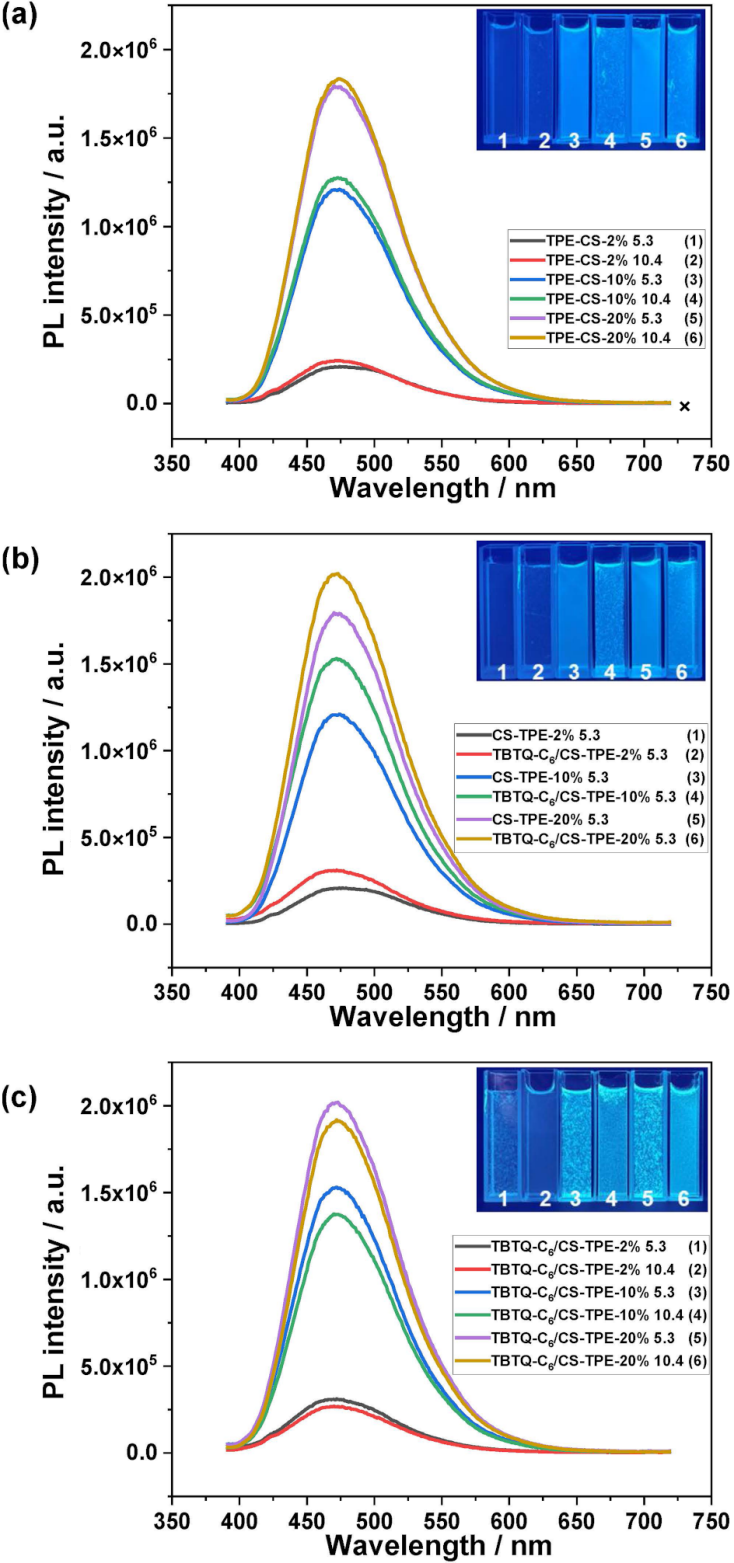
(a) Fluorescence spectra of **CS-TPE** with different *R*_f_ in aqueous solutions at pH 5.3 and 10.4; (b) fluorescence spectra of **CS-TPE** and **TBTQ-C_6_/CS-TPE** with different *R*_f_ in aqueous solutions at pH 5.3; (c) fluorescence spectra of **TBTQ-C_6_/CS-TPE** with different *R*_f_ in aqueous solutions at pH 5.3 and 10.4. Inset: the fluorescent images of the corresponding measured solutions taken under UV irradiation. ([**TBTQ-C_6_**] = 0.10 mM, [**CS-TPE-2%**] = 32 μg/mL), [**CS-TPE-10%**] = 34 μg/mL, [**CS-TPE-20%**] = 35 μg/mL, [**TBTQ-C_6_/CS-TPE-2%**] = 48 μg/mL, [**TBTQ-C_6_/CS-TPE-10%**] = 68 μg/mL, [**TBTQ-C_6 _/CS-TPE-20%**] = 60 μg/mL).

**Fluorescence properties of CS-TPE and TBTQ-C_6_/CS-TPE in water.** The fluorescence properties of **CS-TPE** and **TBTQ-C_6_/CS-TPE** with three different *R*_f_ in aqueous medium were investigated by fluorescence spectrophotometry. The fluorescence emission of **CS-TPE** was significantly enhanced with the increase of its *R*_f_/DL (Figure 7a). This may be caused by the increased labelling of hydrophobic TPE fluorogens, reducing the affinity of the CS chains to the aqueous medium and resulting in the entanglement or wrapping of the TPE fluorogens by polymer chains. As a result, the RIR process was enhanced, greatly increasing the fluorescence. In addition, the fluorescence intensity of **CS-TPE** with the same *R*_f_/DL was slightly enhanced when the pH was increased from 5.3 to 10.4. This is attributed to the lack of protonated amino groups which reduces the hydrophilicity of **CS-TPE** under alkaline conditions and leads to the entanglement and accumulation of **CS-TPE** chains into blocky structures. As shown in Figure 7b, the emission of all **CS-TPE** in a solution at pH 5.3 was enhanced after the addition of the host **TBTQ-C_6_**. This effect can be attributed to the further inhibition of the intramolecular rotation of the phenyl rings of TPE after binding with the host molecule [31]. The fluorescence of the **TBTQ-C_6_/CS-TPE** complex under alkaline conditions was also investigated. When the pH of the solution of **TBTQ-C_6_/CS-TPE** complex was increased from 5.3 to 10.4, the emission was slightly weaker as compared to the solution of **CS-TPE** alone (Figure 7c). This difference may result from the improved water solubility of the supra-amphiphile due to the highly water-soluble behavior of **TBTQ-C_6_** under alkaline conditions in its carboxyl anion form. As a result, the polymer chains were extended, releasing the entanglement and wrapping of the TPE fluorogens, thereby reducing the emission of the fluorogens. The results demonstrate that the fluorescence changes with the pH are tiny for **CS-TPE** alone and also for the **TBTQ-C_6_/CS-TPE** complex, suggesting the stable emission behavior of these nanoparticles, which may have potential applications for biological tracing [25].

## Conclusion

In conclusion, we successfully designed and synthesized different degrees of TPE-labelled CS polymer compounds that exhibit the AIE fluorescence effect and we constructed pH-responsive fluorescent supramolecular spherical nanoparticles based on **CS-TPE** alone and the host–guest **TBTQ-C_6_/CS-TPE** supra-amphiphile. The critical aggregation concentrations of the spherical nanoparticles were determined by transmittance measurements, and the optimal mixing ratios of **TBTQ-C_6_/CS-TPE** were determined. Both types of spherical nanoparticles exhibited good pH responsiveness, i.e., they formed uniform-sized nanospheres at pH 5.3 and collapsed at pH 10.4. Interestingly, when the host molecule **TBTQ-C_6_** was added, the spherical nanoparticles showed significantly less sample build-up and improved dispersion after disintegration under alkaline conditions as compared to **CS-TPE** alone. The fluorescence intensity of both nanospheres was significantly enhanced by increasing the feed ratio/labelling degree due to the unique AIE effect of the TPE fluorescent moiety. Moreover, the fluorescence intensity was significantly enhanced with introducing the **TBTQ-C_6_** host molecule than when **CS-TPE** was present alone. In addition, the fluorescence of both spherical nanoparticles remained relatively stable under different pH conditions. The unique properties of these two fluorescent supramolecular spherical nanoparticles may be valuable for applications in oral drug delivery materials, biomarkers, etc.

## Experimental

**Material preparation.** Chitosan with a degree of deacetylation greater than 95% (viscosity 100, 200 mPa·s) was purchased from Aladdin. All other reagents and solvents were purchased from Xilong Science as analytical grade and were used without further purification. **TBTQ-C_6_** and **TPE-CHO** were synthesized and purified according to the previously reported procedures [16,23]. The ^1^H NMR spectra were recorded by use of a Bruker AV 400 spectrometer in D_2_O and CDCl_3._

**Synthesis of CS-TPE.** The **TPE-CHO** [23] (2 mol %, 10 mol %, and 20 mol %) solution in THF (20 mL) was added to the CS (143.1 mg, 0.9 mmol) solution in 1% glacial acetic acid (30 mL), and the mixture was heated at 70 °C with stirring for 6–10 h. The resulting mixture was then allowed to stand overnight, and the solvent was removed under reduced pressure to obtain the crude Schiff base as a pale-yellow solid. Without further purification, the Schiff base was dispersed in methanol (60 mL) with vigorous stirring for 30 min, followed by slow addition of sodium borohydride (40 mg, 1.1 mmol). The mixture was stirred at room temperature for 24 h and then quenched with ice water. The precipitate was collected by suction filtration, washed with acetone (3 × 100 mL), and dried under vacuum to give **CS-TPE** as a pale-yellow solid. The **CS-TPE** obtained with feed ratios (*R*_f_) of 2, 10, and 20 mol % were denominated **CS-TPE-2%**, **CS-TPE-10%**, and **CS-TPE-20%**, respectively. The labelling degree (DL) of **CS-TPE** was calculated by Equation 1 based on the ^1^H NMR data.


[1] $$\text{DL }(\%)=\frac{\text{integration of }H^{ar}/19}{\text{integration of }H^{2}}\times 100$$

**Preparation of TBTQ-C_6_/CS-TPE nanoparticles. CS-TPE** was dissolved in 1% (v/v) acetic acid by stirring. The pH was increased by adding sodium hydroxide solution dropwise until it reached 5.3. The pH value was verified with a pH meter calibrated with two standard buffer solutions. Then, the **TBTQ-C_6_** solution was dropped into the **CS-TPE** solution and left for 1 h to obtain **TBTQ-C_6_/CS-TPE** nanoparticles. The obtained mixture was centrifuged to take the upper transparent layer for subsequent testing.

**Optical transmittance.** A Shimadzu UV-2600 spectrophotometer with an automated digital thermostat was used to measure the optical transmittance in the range of 200 to 800 nm at 25 °C.

**Fluorescence spectroscopy.** The solid-state fluorescence intensities of three **CS-TPE** bioconjugates and their fluorescence intensities at different pH values, as well as those of the three **TBTQ-C_6_/CS-TPE** supramolecular vesicles at different pH values were measured on a high-sensitivity fluorescence spectrometer HORIBA Fluorolog-3.

**TEM experiments.** The morphology and size of **TBTQ-C_6_/CS-TPE** nanoparticles were studied by use of a Talos F200X G2 field emission transmission electron microscope (TEM). The sample solution was dropped on a copper net and then dried under an infrared lamp to prepare a sample for TEM measurement. The morphology of nanoparticles was observed at an accelerating voltage of 200 kV, and high-resolution TEM images were obtained.

**DLS and Zeta potential measurements.** The measured samples were examined by a laser particle size analyzer (Zetasizer Nano ZS90) with a scattering angle of 90° and a wavelength of 636 nm.

## Supporting Information

File 1 Solid-state CP/MAS ^13^C NMR spectra of **CS-TPE**, optical transmittance and concentration-dependent transmittance of **CS-TPE**, Tyndall effect of **CS-TPE** and **TBTQ-C_6_/CS-TPE** and pH-responsive properties of **TBTQ-C_6_/CS-TPE**.
