# Supplementary material for: pH-Responsive fluorescent supramolecular nanoparticles based on tetraphenylethylene-labelled chitosan and a six-fold carboxylated tribenzotriquinacene
Source: Beilstein J Org Chem. 2023 May 8;19:635–45. doi: 10.3762/bjoc.19.45 (PMC10186267; doi:10.3762/bjoc.19.45)
Supplement: File 1 — Solid-state CP/MAS 13C NMR spectra of CS-TPE, optical transmittance and concentration-dependent transmittance of CS-TPE, Tyndall effect of CS-TPE and TBTQ-C6/CS-TPE and pH-responsive properties of TBTQ-C6/CS-TPE. [file Beilstein_J_Org_Chem-19-635-s001.pdf]

BEILSTEIN JOURNAL OF ORGANIC CHEMISTRY

Supporting Information

for

pH-Responsive fluorescent supramolecular nanoparticles based on tetraphenylethylene-labelled chitosan and a six-fold carboxylated tribenzotriquinacene

Nan Yang, Yi-Yan Zhu, Wei-Xiu Lin, Yi-Long Lu and Wen-Rong Xu

Beilstein J. Org. Chem. **2023,** *19,* 635–645. doi:10.3762/bjoc.19.45

Solid-state CP/MAS ^{13}C NMR spectra of CS-TPE, optical transmittance and concentration-dependent transmittance of CS-TPE, Tyndall effect of CS-TPE and TBTQ-C_6/CS-TPE and pH-responsive properties of TBTQ-C_6/CS-TPE

License and Terms: This is a supporting information file under the terms of the Creative Commons Attribution License (https://creativecommons.org/licenses/by/4.0). Please note that the reuse, redistribution and reproduction in particular requires that the author(s) and source are credited and that individual graphics may be subject to special legal provisions.
The license is subject to the *Beilstein Journal of Organic Chemistry* terms and conditions: (https://www.beilstein-journals.org/bjoc/terms)

Table of contents

Figure S1: Solid-state CP/MAS ^{13}C NMR spectra of CS and CS-TPE S2

Figure S2: Optical transmittance and concentration-dependent transmittance of **CS-TPE** S3

Figure S3: Tyndall effect of **CS-TPE**.. S4

Figure S4: Tyndall effect of **TBTQ-C$_6$/CS-TPE**.. S4

Figure S5: pH-responsive properties of **TBTQ-C$_6$/CS-TPE-2%** S5

Figure S6: pH-responsive properties of **TBTQ-C$_6$/CS-TPE-20%** S6

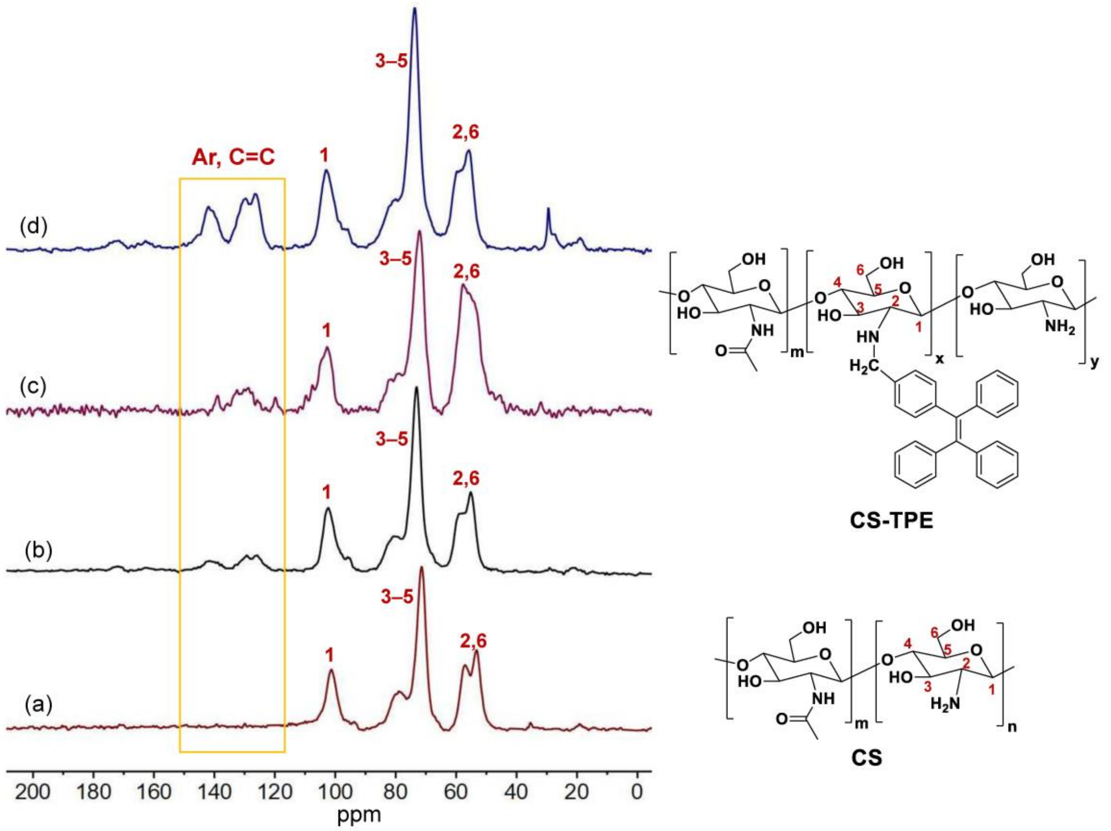

Figure S1: Solid-state CP/MAS ^{13}C NMR spectra of (a) CS, (b) **CS-TPE-2%**, (c) **CS-TPE-10%** and (d) **CS-TPE-20%**.

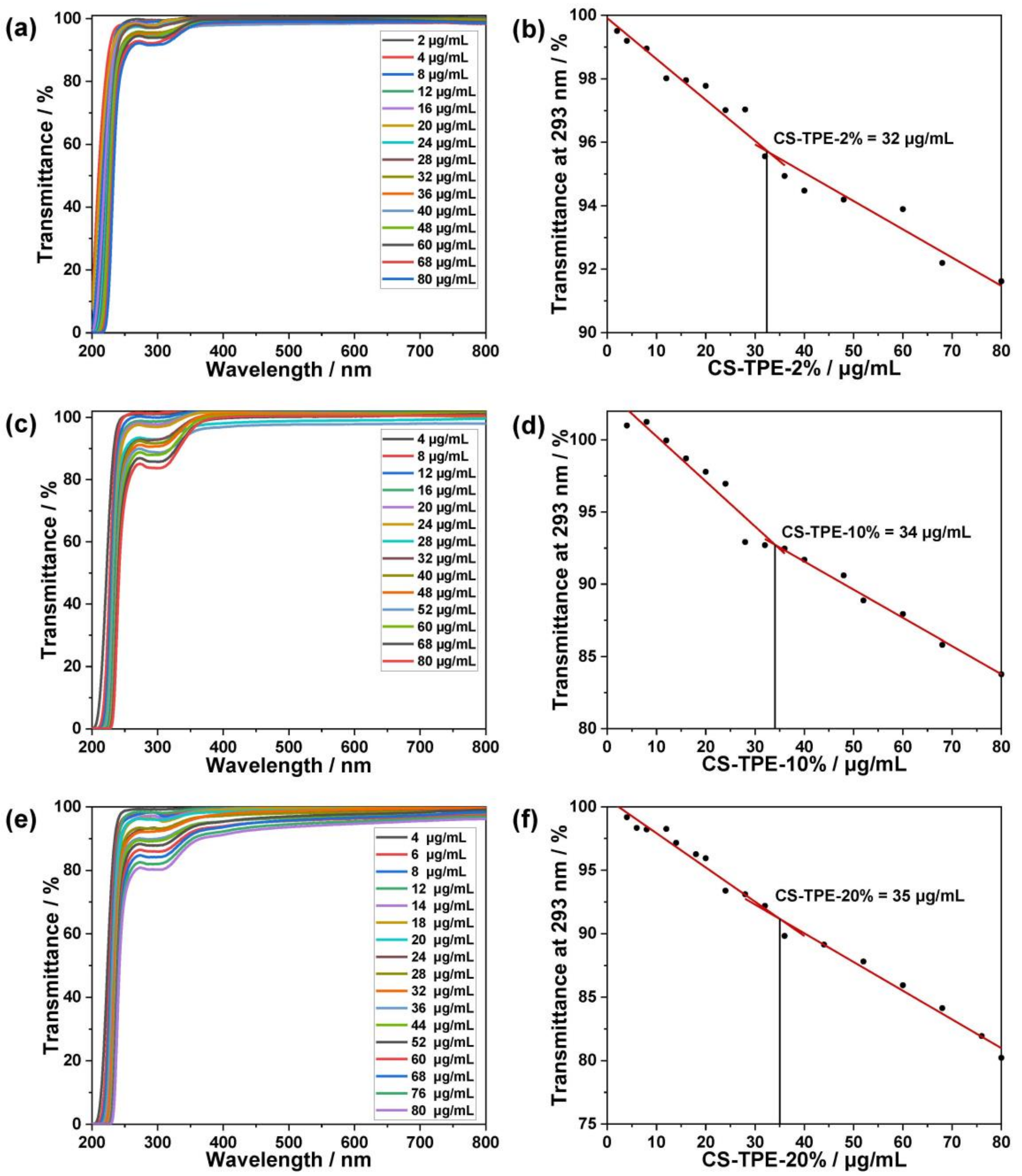

Figure S2: (a) Optical transmittance and (b) concentration-dependent transmittance at 293 nm of **CS-TPE-2%**; (c) Optical transmittance and (d) concentration-dependent transmittance at 293 nm of **CS-TPE-10%**; (e) Optical transmittance and (f) concentration-dependent transmittance at 293 nm of **CS-TPE-20%** in aqueous solutions (pH 5.3).

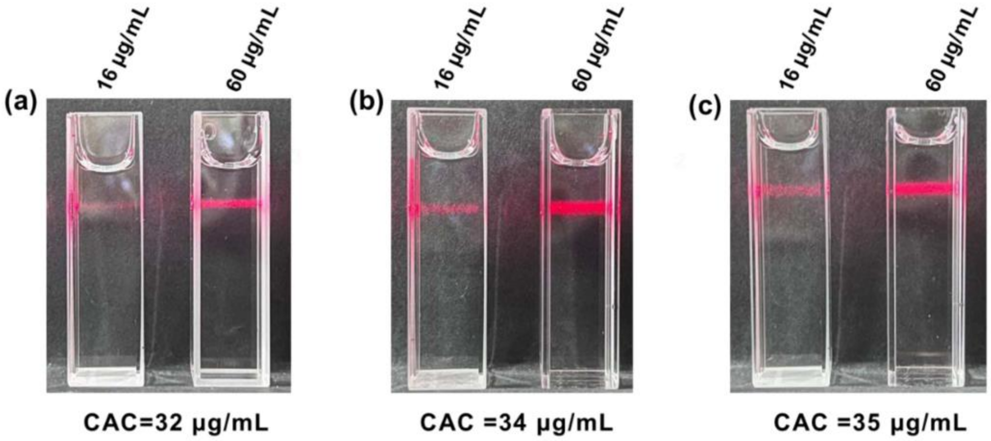

Figure S3: Tyndall effect of (a) **CS-TPE-2%**, (b) **CS-TPE-10%** and (c) **CS-TPE-20%** at concentrations below and above the CACs in aqueous solutions (pH 5.3).

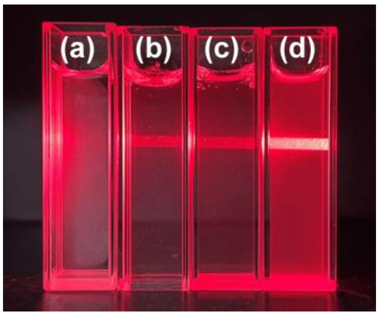

Figure S4: Tyndall effect of (a) **TBTQ-C_6**, (b) **TBTQ-C_6/CS-TPE-2%**, (c) **TBTQ-C_6/CS-TPE-10%** and (d) **TBTQ-C_6/CS-TPE-20%** in aqueous solution at pH 5.3 ([**TBTQ-C_6**] = 0.10 mM, [**CS-TPE**] = 10 μg/mL).

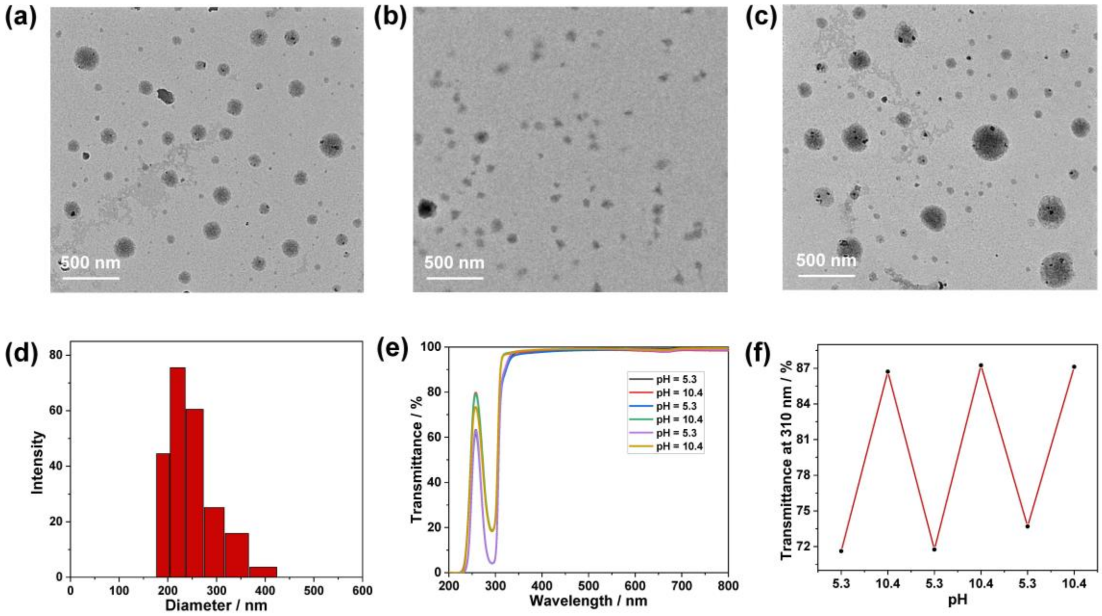

Figure S5: TEM images of (a) **TBTQ-C_6/CS-TPE-2%** in aqueous solution at pH 5.3; (b) after adjustment of the solution (a) to pH 10.4; (c) after readjustment of solution (b) to pH 5.3; (d) DLS data of **TBTQ-C_6/CS-TPE-2%** assembly; (e) Optical transmittance and (f) dependence of the optical transmittance at 310 nm of the **TBTQ-C_6/CS-TPE-2%** assembly under acidic and alkaline conditions over several cycles ([**TBTQ-C_6**] = 0.10 mM, [**CS-TPE-2%**] = 48 μg/mL).

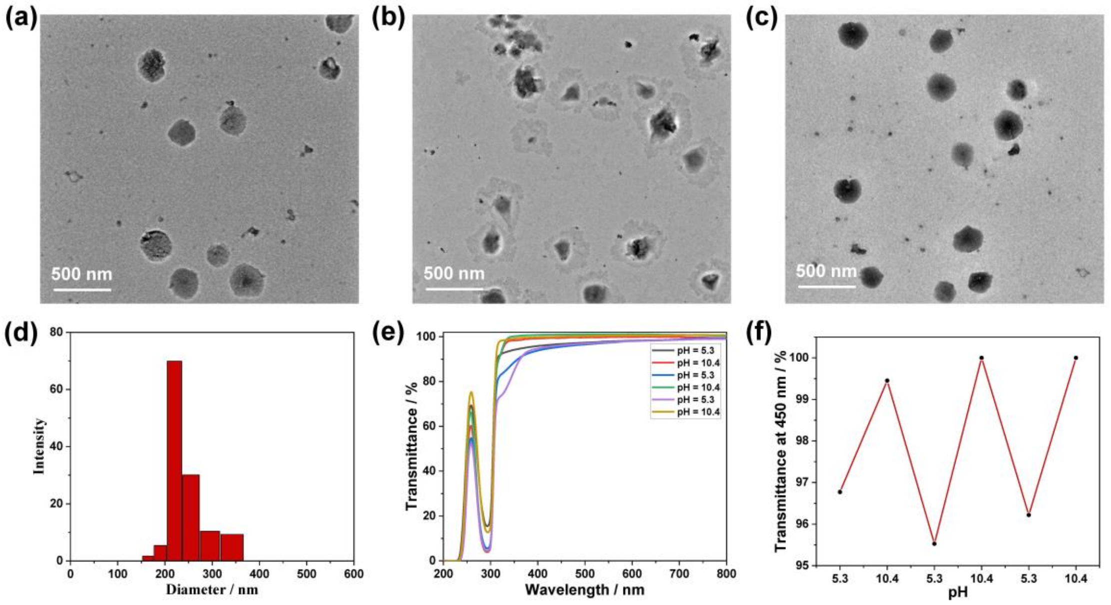

Figure S6: TEM images of (a) **TBTQ-C6/CS-TPE-20%** in aqueous solution at pH 5.3; (b) after adjustment of the solution (a) to pH 10.4; (c) after readjustment of solution (b) to pH 5.3; (d) DLS data of **TBTQ-C6/CS-TPE-20%** assembly; (e) optical transmittance and (f) dependence of the optical transmittance at 450 nm of the **TBTQ-C6/CS-TPE-20%** assembly under acidic and alkaline conditions over several cycles ([**TBTQ-C6**] = 0.10 mM, [**CS-TPE-20%**] = 60 μg/mL).
